# Age-Related Oral and Para-Oral Tissue Disorders: The Evolving Therapeutic and Diagnostic Potential of Exosomes

**DOI:** 10.3390/dj13030106

**Published:** 2025-02-27

**Authors:** Mohamed Khaled Mohamed Maria, Esraa Mohamed Abdel Moniem, Ahmed Khaled Hanafy, Dina B. E. Farag, Israa Ahmed Radwan, Marwa M. S. Abbass, Sara El Moshy, Dina Rady, Christof E. Dörfer, Karim M. Fawzy El-Sayed

**Affiliations:** 1 Oral Biology Department, Faculty of Dentistry, Cairo University, Cairo 12613, Egypt; mohamed.khaled@dentistry.cu.edu.eg (M.K.M.M.); dina.farag@dentistry.cu.edu.eg (D.B.E.F.); esraa.ahmed@dentistry.cu.edu.eg (I.A.R.); marwa.magdy@dentistry.cu.edu.eg (M.M.S.A.); sarah.mahmoud@dentistry.cu.edu.eg (S.E.M.); dina.radi@dentistry.cu.edu.eg (D.R.); 2 Department of Basic Dental Science, National Research Centre, Cairo 12622, Egypt; em.elmalt@nrc.sci.eg; 3 Oral Biology Department, Faculty of Dentistry, Egyptian Russian University, Badr City 11829, Egypt; ahmed-khaledhanafi@eru.edu.eg; 4 Stem Cells and Tissue Engineering Research Group, Faculty of Dentistry, Cairo University, Cairo 12588, Egypt; 5 Clinic for Conservative Dentistry and Periodontology, School of Dental Medicine, Christian Albrechts University, 24118 Kiel, Germany; doerfer@konspar.uni-kiel.de; 6 Oral Medicine and Periodontology Department, Faculty of Dentistry, Cairo University, Cairo 12613, Egypt

**Keywords:** aging, oral, para-oral, MSCs, exosomes, therapy, diagnosis

## Abstract

This review highlights the key molecular and cellular mechanisms contributing to aging, such as DNA damage, mitochondrial dysfunction, telomere shortening, protein dysfunction, and defective autophagy. These biological mechanisms are involved in various oral health conditions prevalent in the elderly, including periodontal disease, oral cancer, xerostomia, dental caries, and temporomandibular joint disorders. Exosomes generated by mesenchymal stem cells possess substantial therapeutic potential. These exosomes are nanosized extracellular vesicles derived from cells and are involved in essential intercellular communication and tissue homeostasis. The exosome-based therapies proved superior to traditional cell-based approaches, due to lower immunogenicity, ease of storage, and avoidance of complications associated with cell transplantation. Furthermore, the diagnostic potential of exosomes as non-invasive biomarkers for aging processes and age-related oral diseases offers insights into disease diagnosis, staging, and monitoring. Among the challenges and future perspectives of translating exosome research from preclinical studies to clinical applications is the need for standardized procedures to fully harness the therapeutic and diagnostic capabilities of exosomes.

## 1. Introduction

Aging is the loss of cellular physiological function over time with the accumulation of tissue damage. This leads to physiological dysfunction of organs, diminished adaptability and resistance, and increased probability of death [1]. The percentage of persons over 60 is expanding globally, and it is predicted that by 2050, the percentage will nearly double, growing from 12% to 22% [2,3,4]. Numerous biological pathways have been put forward as contributing to the aging process. However, because there are multiple interactions between these various systems, currently, no single mechanism can fully account for aging [5,6].

Among the involved mechanisms at a molecular level is DNA damage due to replication errors, reactive oxygen species (ROS), and external factors like radiation or ultraviolet light, which occur concomitantly with decreased efficiency of DNA repair and ROS elimination with age, leading to accumulated damage and mutations that result in cellular dysfunction [7]. Epigenetic changes such as DNA methylation and histone modification also contribute to aging phenotypes and disorders [8]. Mitochondrial dysfunction, marked by decreased respiratory capacity per mitochondrion, is a defining feature in aging tissues and senescent cells, increasing ROS production and causing further cell damage and aging [2,9]. Telomeres, which protect chromosome ends, shorten over time due to reduced telomerase activity, inducing cell cycle arrest and inflammation, thus contributing to aging [10,11]. Protein homeostasis declines with age, disrupting processes like synthesis and degradation, leading to age-related diseases [12,13]. Autophagy activity diminishes with age across species, reducing lysosomal protein hydrolysis and promoting cellular damage and age-related illnesses [14].

**Figure 1:**
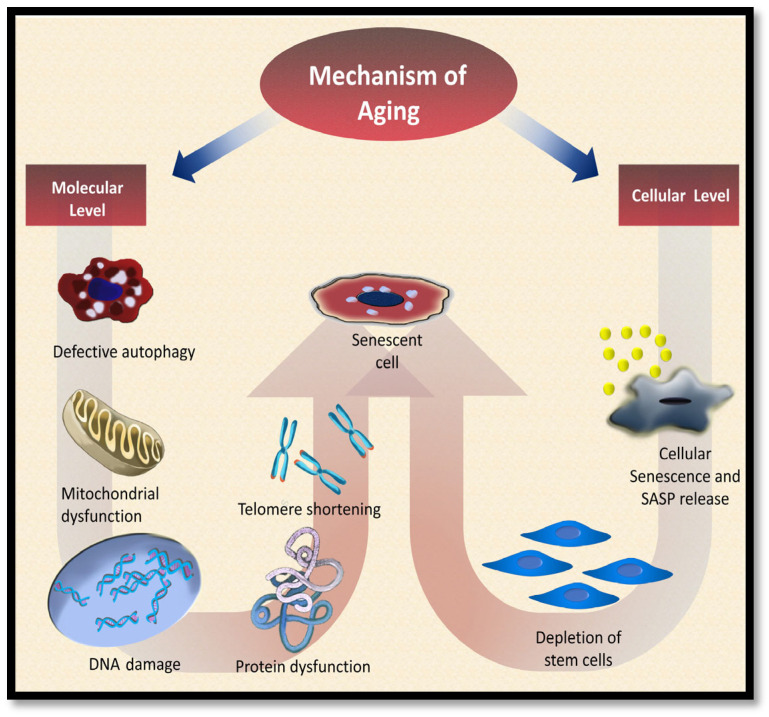
The mechanisms associated with aging at molecular and cellular levels.

At a cellular level, senescence arises from triggers like DNA damage and oxidative stress, where damaged cells accumulate due to decreased clearance rates, affecting tissue function through secretory phenotypes [15]. Stem/progenitor cell depletion and impaired function reduce regenerative capacity, accelerating tissue malfunction and senescence [16]. Aging also enlarges stem/progenitor cells, decreasing proliferation and altering metabolism, impairing overall stem cell functionality [17]. These molecular and cellular changes as summarized in Figure 1. They disrupt homeostasis, reduce regeneration potential, cause low-grade inflammation, and impair intercellular communication, accelerating aging and contributing to related disorders [18].

These molecular and cellular alterations contribute to the pathogenesis of various age-related diseases, including those affecting oral and para-oral tissues [2]. Oral conditions such as periodontal disease, xerostomia, dental caries, temporomandibular joint (TMJ) disorders, and oral cancer disproportionately afflict the elderly, significantly impairing quality of life [19,20]. Traditional therapeutic approaches for these conditions often face limitations, including incomplete efficacy, invasiveness, and systemic side effects [21], necessitating innovative strategies to address the unique challenges of aging tissues [22].

In recent years, extracellular vesicles, particularly exosomes derived from mesenchymal stem cells (MSCs), have emerged as promising tools in regenerative medicine and diagnostics. Exosomes are nanosized vesicles that mediate intercellular communication by transferring bioactive cargo—such as proteins, lipids, and nucleic acids—to recipient cells [23]. Compared to cell-based therapies, MSC-derived exosomes offer distinct advantages, including reduced immunogenicity, ease of storage, and avoidance of risks associated with direct cell transplantation. Preclinical studies highlight their capacity to modulate inflammation, promote tissue regeneration, and restore homeostasis in aging-related pathologies [24]. Furthermore, their presence in bodily fluids and disease-specific molecular signatures position exosomes as non-invasive biomarkers for early diagnosing and monitoring oral diseases. Despite these advancements, critical challenges persist in translating exosome-based therapies and diagnostics into clinical practice. These include standardization of isolation protocols, production scalability, and understanding the precise mechanisms underlying their therapeutic effects [25].

This review aims to comprehensively analyze the therapeutic and diagnostic applications of MSC-derived exosomes in aging-related oral and para-oral diseases, elucidate their molecular mechanisms of action, and highlight challenges in transitioning these findings from preclinical research to clinical practice. By integrating existing knowledge and identifying gaps, this work provides a foundation for future research to optimize exosome-based strategies to improve oral health outcomes in the elderly.

## 2. Oral and Para-Oral Disorders Related to Aging

Geriatric patients have a high incidence of age-related diseases, where 90% of people above 65 years of age are reported to have at least one of them, with 75% having two or more diseases related to age [16,26]. Consequently, it was proposed to the World Health Organization that aging should be acknowledged and incorporated as a disease in the International Classification of Diseases 11th Revision (ICD-11) to facilitate treatment and prevent or postpone the onset of chronic non-communicable diseases. The proposal received approval in 2019, set to take effect in 2022 [27,28,29,30].

According to the Global Burden of Disease, Injury and Risk Factor Study, oral disease affects over 280 million older persons aged 70 and over, and is ranked as the 22nd most common cause of disability-adjusted life-years worldwide, affecting over 280 million older persons aged 70 and over [31].

**Figure 2:**
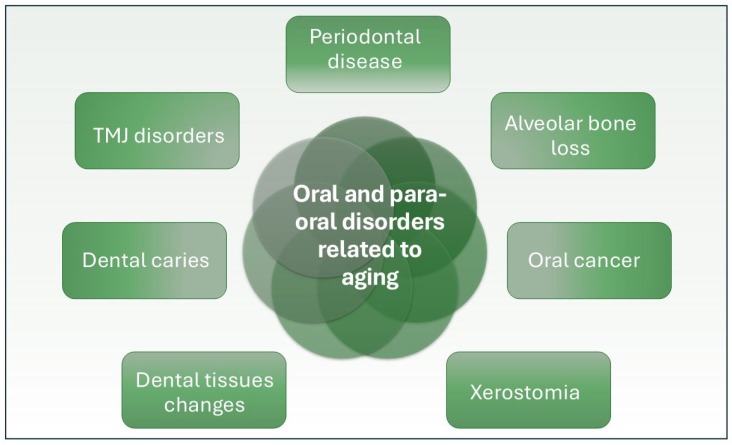
Oral and para-oral disorders related to aging.

Advanced age is the primary determinant for a range of prevalent oral health disorders, including periodontal disease, oral cancer, dental caries, and TMJ disorders [32]. Other oral conditions to be found in older people are oral candidiasis, erythematous lesions, and angular cheilitis [33]. Multiple age-related oral disorders exhibit molecular connections with various key features of aging, such as genomic instability, epigenetic modification, telomere shortening, cellular aging, and altered intercellular communication [33], as presented in Figure 2. 

Periodontitis is a complex multifactorial chronic inflammatory oral disease associated with a dysbiosis of the oral microbiome [34]. It is typically caused by a microbial challenge with a subsequent intense chronic inflammatory process that progressively destroys the tooth-supporting system consisting of the periodontal ligament, cementum, and alveolar bone. It can result in tooth loss [35,36]. Periodontal disease is a prevalent oral health problem that commonly affects older people [37]. The incidence of periodontal diseases often rises from 5% to 80% between the ages of 15 and 60 [38]. A patient’s susceptibility to periodontal disease, as well as their responsiveness to therapy, may be affected by age-related immunologic alterations and histological changes in the periodontal tissue [39,40]. The link between periodontal diseases and cardiac disorders [41], diabetes [42], and osteoporosis [43] should be considered in the same context. Because of these factors, it is undeniable that periodontal disease prevention, diagnosis, and treatment are crucial for preserving general health as an individual age.

Moreover, age-related jawbone atrophy is thought to be primarily caused by periodontitis, tooth loss, the duration of edentulism, the pressure from wearing dentures, and osteoporosis are also believed to be contributing factors [44,45]. Alveolar bone loss can cause tooth loss reciprocally. The patient’s ability to eat may be hampered by tooth loss and alveolar bone atrophy, restricting their options for food and jeopardizing their nutrition [46].

As the most prevalent type of cancer of the head and neck, oral cancer carries a high risk of both morbidity and mortality [47]. It can affect the tongue, cheek lining, hard and soft palates, throat, and oral cavity floor. Oral cancer claims the lives of 11,000 people year, with over 54,000 new cases reported [48]. According to the literature, individuals aged 45 to 64 years had a higher incidence of oral cancer than those from 15 to 19 years old [49]. The increased incidence of oral cancer among elderly individuals is believed to be attributed to age-related deterioration in the defensive salivary antioxidant mechanisms and/or age-related amplification in the severity of oral carcinogen assaults, such as those induced by reactive nitrogen species (ROS), resulting in DNA aberrations [50].

Furthermore, cancer patients can experience both acute and chronic oral toxicities due to cancer treatment, such as mucositis, xerostomia, and salivary gland dysfunction (SGD) [51]. Xerostomia or dry mouth in cancer patients can be a result of chemotherapy and radiation therapy to the head and neck [52]. Radiotherapy can lead to dry mouth as a result of indirect damage to the gland’s epithelial and connective tissues, which includes blood vessels and nerves [51]. In patients with head and neck cancer receiving radiotherapy, irreversible damage to salivary glands is found in 63–93% of cases [53], and the impairment is related to the dosage; when doses of 40–50 Gy are given, up to 75% of parotid gland function might be compromised [54]. Dry mouth due to chemotherapy is common in 10–80% of patients in treatment, irrespective of the cancer type [55]. Oral symptoms typically start between the 7th and 10th day following chemotherapy treatment and subside after chemotherapy concludes, in contrast to radiotherapy-induced SGD, which has long-lasting and often permanent effects on SGD [53]. Symptoms consist of, but are not restricted to, dry oral mucosa leading to oral pseudomembranous candidiasis, halitosis, oral dysesthesia, decreased taste sensation, and challenges in chewing, swallowing, and speaking [55].

Regardless cancer treatment, older adults frequently experience xerostomia, and its frequency rises with age [56]. Thirty percent of patients over 65, and up to 40 percent of patients over 80, suffer from xerostomia [57]. According to Smith et al., older adult groups (>70 years) had salivary flow rates almost half as high as those of the 20-to-30- and 40-to-50-years groups [58]. Sjögren syndrome, especially in females, is the most significant of the several systemic diseases that can impair salivary function in older people [59]. Saliva quantity and quality deficiencies could, in turn, have detrimental effects on dental and oral health. Since saliva is required to prepare food for digestion, patients with salivary hypofunction frequently complain of taste disruption, trouble masticating, and difficulty swallowing [60].

Several changes in dental tissues are thought to be typically related to aging. These changes include wearing of the enamel with subsequent exposure to the dentin, which in turn will subsequently wear more rapidly than enamel. A study of over 700 older people found that over 85% of individuals in all groups were affected by tooth wear [61]. Moreover, the 2009 ADHS illustrates how common the matter is, as a total of 95% of dentate individuals aged 75–84 exhibit wear, with 44% and 6%, respectively, displaying moderate or severe wear [62]. Pulp calcification, reduced pulp dimensions, decreased stem/progenitor cell density, and increased cellular senescence are among the pulpal changes found during aging [63], which results in reduced pulp self-regenerative capability [64].

Additionally, high incidence rates of coronal and root surface caries have been found among older individuals worldwide. Patients over 60 years of age have a root caries incidence that is twice that of patients under 30 years, with persons over 80 years having a root caries incidence of 64% and a coronal caries incidence of up to 96%. Age-related oral disorders such as periodontal disease, oral cancer, and xerostomia are possible contributors to dental caries in older people. Periodontal disease through affecting the supporting structures of teeth, can lead to tooth mobility and exposure of tooth surfaces, making them more susceptible to dental caries [60,65,66].

The TMJ is the most complex joint that attaches the mandibular condyle with the temporal bone articular surface [67]. Degenerative disorders of the TMJ are the second most prevalent chronic musculoskeletal disorder. In affected people, this could lead to acute pain and limited joint mobility [68]. Advancing in age is a notable risk factor for the occurrence of degenerative illnesses in the TMJ. The frequency of TMJ dysfunction symptoms and radiographic alterations in condylar morphology were 18.6% and 81.3%, respectively, between the ages of 20 and 60 [69]. The musculoskeletal health of an individual, including the osteochondral tissues of the TMJ, deteriorates as they age [70]. Degenerative changes, such as condylar resorption, condylar sclerosis, and subcondylar cysts, are frequently observed and are more prevalent in individuals aged 50 to 79 [71].

## 3. Cell-Free Therapy Using Exosomes for Aging

The development of therapeutic strategies against aging, including age-related oral and para-oral diseases, is necessary to improve oral health and quality of life in the elderly. MSCs are multipotent stem/progenitor cells that can differentiate into multiple lineages, playing a crucial role in a wide range of clinical applications [72]. Various types of MSCs, including bone marrow stromal stem cells (BM-MSCs), adipose-derived stromal cells (ADSCs), dental pulp stem cells (DPSCs), dental follicle stem cells (DFSCs), stem cells from human exfoliated deciduous teeth (SHEDs), stem cells from the apical papilla (SCAP), periodontal ligament stem cells (PDLSCs), alveolar bone proper-derived stem cells, and gingival stem cells revealed significant therapeutic potentials in regenerative medicine [73]. Thanks to their immunomodulatory and anti-inflammatory characteristics, there is a growing interest in studying the nature, behavior, and possible therapeutic potentials of these types of cells [74,75].

Stem cell-based therapies show significant potential for oral and para-oral disorders, but the majority of treatments remain in experimental or initial clinical trial stages. Periodontal regeneration [76,77] and craniofacial bone reconstruction [78,79] are the most advanced toward clinical use, whereas salivary gland regeneration [80] and TMJ repair [81] remain in preliminary trials. Research on oral cancer emphasizes targeting cancer stem cells instead of employing stem cells for direct treatment [82].

The mechanism by which MSCs exert their therapeutic effects does not solely involve direct cellular engraftment and replacement of damaged tissues. Instead, the paracrine effectors released by these stem/progenitor cells are considered a chief factor in fulfilling this role. As a result, cell-free therapy is believed to be a novel alternative that outweighs cell-based therapy [83]. MSCs exert their paracrine action by releasing a variety of substances or molecules into the extracellular environment. Secreted factors encompass a variety of substances, such as soluble proteins, free nucleic acids, lipids, and extracellular vehicles (EVs). EVs can be categorized into apoptotic bodies, microparticles or microvesicles (MVs), and exosomes, which vary in terms of their origin, release mechanism, size, composition, and purpose [84]. Apoptotic bodies are believed to be released from dying cells directly into the extracellular compartment. According to literature, their diameters could vary from 50 nm to 5000 nm, with most apoptotic entities tending to be greater in size [85]. When a cell contracts, higher hydrostatic pressure causes the plasma membrane of the cells to separate from the cytoskeleton, forming these unique bodies [86]. Apoptotic bodies could possess intact organelles, chromatin, and low glycosylated protein content; therefore, elevated amounts of proteins related to the nucleus, mitochondria, golgi apparatus, endoplasmic reticulum, and other organelles are expected to be evident [87].

MVs as a subtype of EVs arise via an outward budding from plasma membrane of cells, where their size usually ranges from 100 nm to 1 μm [88]. Due to its release pattern, MVs are easily understood to possess cytosolic and plasma membrane-associated proteins, particularly those that tend to accumulate on the surface of the plasma membrane, including tetraspanins [89]. In addition, heat shock proteins, integrins, and cytoskeletal proteins have all been found to be present in MVs [90]. Prior to the discovery that MVs are engaged in cell-to-cell communication between nearby and distant cells, it was believed that MVs were a dumping mechanism used by the cell for clearance of undesirable substances [91].

### 3.1. Exosomes

Exosomes are extracellular organelles that are nanosized and derived from cells. Their diameter typically ranges from 30–150 nm. Such vesicles are surrounded by a lipid bilayer, packed with various contents derived from the mother cell, and released into the extracellular compartment. Exosomes that have been secreted may be identified in several bodily fluids, such as saliva, plasma, and urine. Exosomes are primarily regarded as having a significant role in intercellular communication and serve as a vehicle for transporting contents to recipient cells. The composition of exosomes is specific to the originating cell. They can carry signals from the parent cell to surrounding or target cells without the need for direct cell-to-cell contact [92,93].

Exosomes generated from MSCs demonstrate significant promise as cell-free therapeutic agents in the field of regenerative medicine. Exosomes formed from MSCs possess the characteristics of the cells they originate from and can enhance cell self-repair, regenerate tissue, and restore tissue balance [94,95]. Exosomes derived from MSCs are believed to be the primary effective paracrine component, exerting biological effects that are nearly identical to those of whole MSCs [96,97]. Exosome-dependent therapies additionally possess a greater safety profile and uncomplicated storage without compromising function, and avoid probable clinical complications such as pulmonary embolism after MSC intravenous infusion. Additionally, the risk of uncontrolled cell growth, teratoma formation, and toxicity related to repeated injection is eliminated [83].

#### 3.1.1. Exosomes Biogenesis

The process of exosome biogenesis begins with early endosomes, when the endosomal membrane undergoes inward budding to create intraluminal vesicles within multivesicular bodies (MVBs) [98,99]. After merging with the plasma membrane, certain intraluminal vesicles are eliminated into the extracellular environment; the majority fuse with lysosomes for later destruction [100]. Although the direct sprouting of the plasma membrane is believed to contribute significantly to exosome biogenesis, the endosome-dependent pathway remains widely regarded as the principal mechanism for exosome formation [101,102]. The MVBs fuse with the plasma membrane to form exosomes, which are then secreted extracellularly [101]. The fusion process is mediated by numerous mechanisms, including Ras-associated binding (Rab) GTPases [103]. Rab GTPases are extensively preserved regulators of vesicular transport, and the human genome contains a total of 66 members belonging to this family. Their activity alternates between an active and inactive state, regulated by guanine nucleotide exchange factor and GTPase-activating protein. They operate through downstream molecules, including coat proteins and motor proteins, to initiate downstream membrane trafficking. Rab GTPases govern many trafficking pathways and execute distinct functions in a sequence of membrane trafficking processes. Rab GTPases play a crucial role in ensuring the accurate delivery of cargo to their intended destinations. They achieve this by regulating the generation of membrane buds, facilitating vesicular transport via the cytoskeleton, and promoting the fusing of membranes with the target compartment [104].

After being transported and docked to the plasma membrane, secretory MVBs connect to the soluble N-ethylmaleimide-sensitive component attachment protein receptor membrane fusion machinery. Exosomes are released into the extracellular space and then interact with the extracellular matrix (ECM), exerting an influence on nearby cells. They can be taken up by other cells through direct fusion of their membranes, through endocytosis, or cell-specific phagocytosis. Alternatively, they can enter the bloodstream and exert their paracrine effect [105].

#### 3.1.2. Composition of Exosomes

Exosome contents not only reflect the mother cell’s composition, but also reflect a regulated protein-sorting mechanism linked to the process of exosome formation and/or the loading of its contents [106]. The content is composed of a collection of proteins, including receptors, transcription factors, enzymes, lipids, ECM proteins, and nucleic acids, including messenger RNA (mRNA) and microRNA (miRNA) [107]. The protein composition analysis of exosomes indicates that certain proteins are unique to the cell and tissue of origin, while others are present in all exosomes [108]. The classic examples of specific exosome proteins include adhesion molecules, such as cell adhesion molecules, integrins, tetra-spanins, and major histocompatibility complex class I and II, which are present on the surface of B lymphocytes and dendritic cells, as well as transferrin receptors on the surface of reticulocytes. On the contrary, a wide variety of non-specific proteins are frequently reported, including heat shock (70 and 90), transferring proteins such as Rab2, and cytoskeleton proteins such as actin and myosin [109].

Exosome cargo also contains a variety of proteolytic enzymes, the majority of which are related to the metalloproteinase (MMPs) group, including membrane-anchored MMPs, disintegrin, and MMPs without or with thrombospondin motifs (ADAMs and ADAMTS, peptidases, respectively) [110]. ADAMTS is a multidomain enzyme that acts extracellularly due to the absence of a lipid bilayer anchor. At the same time, ADAMs are transmembrane polypeptides that have a unique potential to combine adhesion, proteolysis, and signaling in addition to involvement in a variety of cellular functions. It was suggested that ADAMs and ADAMTS peptidases might be responsible for glycosidases present within the exosomes. Even though such an enzyme family has not yet been thoroughly investigated in exosomes, there is a growing scientific suggestion that they can actively carry on the proteolysis process either located on the outer surface of the cell or within the ECM [111].

Additionally, the lipid composition of exosomes is exceedingly cell specific. Lipids are essential for the regulation of homeostasis in recipient cells, as well as the protection of exosome structure and exosome biogenesis [112]. Cell membranes and exosomes frequently include several lipids, including sphingomyelin, phospholipids, ganglioside GM3, and cholesterol. Nevertheless, the proportion of these lipids found in exosome membranes can differ based on the specific kind of cell producing the exosome, the physiological state of the producing cell, and the ultimate purpose and role of the exosome [113,114,115].

**Figure 3:**
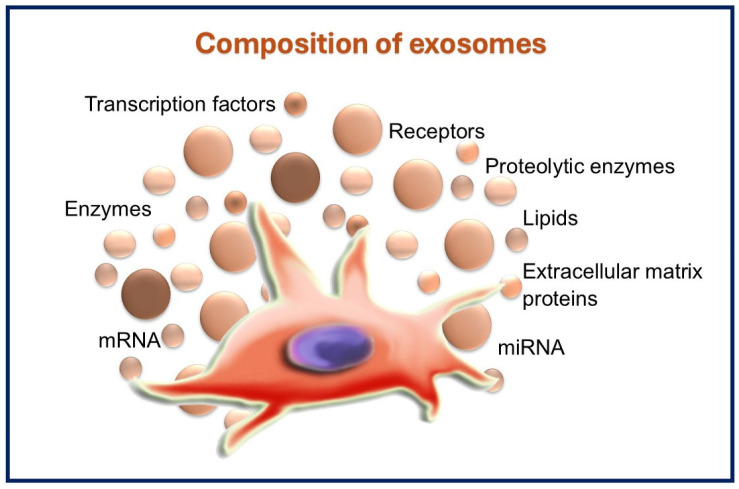
Composition of exosomes.

Nucleic acids, which include mRNA, miRNA, and other noncoding RNAs, were further detected as cargo in stem/progenitor cells exosomes [116] and represent the most numerous exosomal cargo molecules. mRNA is a single-stranded RNA that is involved in the biosynthesis of proteins. mRNA is generated from a DNA template during the transcription process. Protein information can be transmitted from the nucleus of a cell to the targeted cell through the incorporation of mRNA into exosomes [117]. On the other hand, exosomal miRNA has been linked to many human diseases and is being pursued as clinical diagnostic tools and therapeutic targets. They are thought to be endogenous noncoding RNAs that have a significant participation in the protein biosynthesis regulation via post-transcriptional regulation [118]. The composition of exosomes is summarized in Figure 3.

#### 3.1.3. Methods of Exosome Isolation

Among the common methods to isolate exosomes are ultrafiltration, precipitation, and ultracentrifugation, in which exosomes isolated by the ultracentrifugation method exhibit a smaller dimension and more homogenous particles when compared to exosomes isolated by the two other approaches [119]. While the classic methods chiefly comprise differential ultracentrifugation, size-based isolation, and polymer-based precipitation, these methods could be used in specific cases as handling large-capacity samples [120].

On the other hand, recent techniques, including the use of extracellular vesicles on demand chips that could be utilized to extract lung cancer-exosomes [121], microfluidics [122], field flow isolation [123], label-free magnetic isolation [124], and functional micro/nanostructures [125] might be utilized to yield exosomes effectively. Other approaches were introduced, relying on modifications of the classical methods, including dichotomic size-exclusion chromatography [126] and ultracentrifugation together followed by size-exclusion chromatography [127].

#### 3.1.4. Methods of Exosome Characterization

Generally, exosomes can be characterized by different techniques, including transmission electron microscopy, nanoparticle tracking analysis (NTA), and flow cytometry [128]. Characterization methods of exosomes could be classified into quantitative, qualitative, and single-vesicle characterization techniques. Quantitative methods are applied to evaluate the success of exosomes’ isolation, yield, and loads regarding biomolecules including proteins, lipids, and nucleic acids. These quantification methods could be achieved through NTA, dynamic light scattering (DLS), and resistive pulse sensing [129]. However, qualitative exosome characterization methods comprise Western blotting, Raman spectroscopy, and next-generation sequencing [130,131], through which the existence of the wide variety of exosomal constituents are validated. Alternatively, single-vesicle characterization methods, including atomic force microscopy along with DLS, are considered crucial tools in therapeutic applications of exosomes as drug carrier vehicles [132].

### 3.2. Anti-Aging Potential of MSC-Derived Exosomes

Exosomes have been reported to have modulating effects on aging [133]. Treatment of aged mice with young mice exosomes resulted in the reversion of the mice’s aging-associated molecule expression, where p16^Ink4A^, mammalian target of rapamycin (mTOR), and insulin-like growth factor 1 (IGF1) receptors were significantly downregulated [134]. Moreover, it was demonstrated that the usage of MSCs-EVs (including exosomes and MVs) causes a reduction in the production of the pro-inflammatory cytokines interleukin (IL) -6 and prostaglandin E2, as well as a decrease in the oxidative stress of senescent MSCs, with cell growth enhancement and alleviation of aging cellular phenotypes [135].

Adipose-derived stem cell (ADSCs) exosomes were shown to improve skin photodamage in rat photoaged skin, where a reduction in the epidermal thickness and improvement of the dermal thickness were demonstrated seven days after treatment. Furthermore, collagen type I mRNA expression was increased, and type III collagen, MMP-1, and MMP-3 were decreased, proposing that exosomes could be a promising strategy for treating skin that has been damaged by exposure to sunlight [136].

The effect of human placental MSC-derived exosomes was also studied on aged skin in a double-blinded randomized controlled clinical study. The exosome treatment group showed improved skin tone, quality, and clarity compared to the control group (saline/plecebo group), with a reduction in wrinkles, pores, pigmentation, and skin vascularity [137]. Thus, exosomes might serve as a promising approach for age reversion and the treatment of age-related diseases [138].

### 3.3. Therapeutic Potential of MSC-Derived Exosomes in Age-Related Oral and Para-Oral Disorders

Exosome therapy is being explored for different oral conditions because of its regenerative, immunomodulatory, and anti-inflammatory effects. Periodontal diseases, oral cancer, salivary gland conditions (such as Sjögren’s syndrome), TMJ disorders, and craniofacial bone loss exhibit several traits that render them suitable for therapies utilizing exosomes.

These common traits include persistent inflammation which plays a critical role in periodontal diseases [139,140], Sjögren’s syndrome [141], TMJ disorders [142], and bone loss [143], and may also contribute to the advancement of oral cancer [140]. Exosomes, particularly those obtained from MSCs, possess anti-inflammatory and immunomodulatory characteristics that aid in controlling excessive immune reactions [144].

Tissue deterioration and hindered regeneration are frequent traits seen in periodontal diseases [145], TMJ disorders [146], and craniofacial bone loss [147], all of which involve ongoing destruction of tissue and bone. Additional phenomena are the atrophy of salivary glands in Sjögren’s syndrome [148] and the degradation of structural integrity in oral cancer because of tumor advancement [149]. Exosomes facilitate tissue healing, boost cell growth, and encourage angiogenesis, rendering them appealing for regenerative uses [150].

Moreover, fibrosis is a hallmark of TMJ disorders [151], Sjögren’s syndrome [152], and complications following cancer treatment [153]. Exosomes can regulate the remodeling of ECM and safeguard against excessive fibrosis [144].

Elevated oxidative stress plays a role in the development of the mentioned diseases, resulting in cellular damage and apoptosis, similar to salivary gland cell death in Sjögren’s syndrome [154] and osteocyte apoptosis in bone loss [155]. Exosomes possess antioxidant and cytoprotective qualities that can alleviate these impacts [144].

Microenvironmental changes and crosstalk are crucial in the advancement of periodontal diseases and oral cancer, with the surrounding microenvironment comprising immune cells, fibroblasts, and epithelial cells [156,157]. Exosomes aid in cell-to-cell communication, altering the disease microenvironment to promote healing and diminish pathogenic signals [158].

Stem cell depletion and endogenous stem cell impairment, as in osteoarthritis, the capacity for damaged articular cartilage to regenerate is notably compromised due to the limited availability and dysfunction of endogenous MSCs caused by aging and the disease [159]. Similarly, in precursor cells within salivary glands [160], exosome treatment can activate and revive stem cell function [161].

Conditions like periodontitis [162] and TMJ disorders [163] frequently entail diminished blood flow, which aids in the advancement of the disease. Exosomes enhance angiogenesis, facilitating better vascularization and oxygen supply [161].

The common factors—chronic inflammation, tissue degeneration, immune dysregulation, fibrosis, oxidative stress, and vascular impairment—make these conditions ideal candidates for regenerative therapy using exosomes. Exosomes provide a cell-free, low-immunogenic therapeutic method that could influence the disease environment, enhance tissue regeneration, and re-establish homeostasis.

#### 3.3.1. Exosomes and Periodontal Diseases

Exosome-based therapies have been proposed as a novel and potentially effective therapeutic strategy for ameliorating periodontitis and improving bone resorption in the alveoli. Human dental pulp stem cell exosomes (DPSC-Exos) derived from 3D cultures were used in a mouse model of ligature-induced periodontitis. These exosomes were found to restore the balance between T helper (Th)17 cells and T regulatory (Treg) cells, as well as regulate the immunological responses in the inflamed periodontium. This effect was achieved as the 3D-exosomes were enriched with miRNA-1246, which suppresses the expression of the nuclear factor of activated T-cells, a key factor in Th17 cell polarization [164]. Furthermore, DPSC-Exos have shown to possess the capability to direct the transformation of histocytes from a pro-inflammatory state (M1) to an anti-inflammatory state (M2) and to promote the healing of alveolar bone in mice with periodontitis [165]. This effect is likely due to the presence of miRNA-1246 in DPSC-Exos [165] and through their potential to regulate inflammation directly by inhibiting the IL-6/JAK2/STAT3 signaling pathway during acute inflammatory stress [166].

Rat bone marrow mesenchymal stromal cell (BM-MSC) small EVs were found to significantly enhanced the migration, proliferation, and osteogenic differentiation of human periodontal ligament cells (PDLCs) in vitro. BM-MSC-small EVs inhibited osteoclastic activity by downregulating the receptor activator of nuclear factor kappa-Β ligand (RANKL)/osteoprotegerin (OPG) expression ratio, promoted the polarization of macrophages to the anti-inflammatory M2 phenotype, and increased transforming growth factor (TGF)-β1 expression—accordingly, contributing to the inhibition of periodontal inflammation and promoting tissue regeneration in a rat model of periodontitis [167]. Similarly, exosomes derived from human BM-MSCs significantly suppressed the inflammatory response triggered by *Porphyromonas gingivalis* in macrophages. This was evidenced by reduced levels of pro-inflammatory cytokines such as IL-6, IL-1β, and tumor necrosis factor (TNF)-α. The exosomes modulated the immune responses and downregulated genes like MMP-9, chemokine (C-C motif) ligand 7, and leukocyte-specific transcript 1, while upregulating genes related to inflammation resolution, such as chemokine-like receptor 1. Human BM-MSC-Exos were enriched with key miRNAs, including miRNA-100-5p, miRNA-125b-5p, and miRNA-21-5p, which are known to have anti-inflammatory properties. In a rat model of ligature-induced periodontitis, weekly injections of human BM-MSC-Exos into gingival tissues reduced tissue destruction and immune cell infiltration, leading to less alveolar bone loss compared to controls [168].

Exosomes derived from human leukocyte antigen haplotype homo dental pulp cells significantly reduced alveolar bone loss compared to the control group in a ligature-induced periodontitis mouse model and promoted the migration of both human dental pulp cells and mouse osteoblastic cells in vitro [169].

Moreover, the culture microenvironment significantly affects the exosomes’ therapeutic potential, as exosomes derived from normal-glucose-cultured periodontal ligament stem cells (NG-PDLSC-Exos) significantly inhibited osteoclast formation and differentiation in vitro compared to those derived from high-glucose-preconditioned PDLSCs (HG-PDLSC-Exos). In a diabetic periodontitis mouse model, NG-PDLSC-Exos attenuated alveolar bone loss and osteoclastogenesis more effectively than HG-PDLSC-Exos through miRNA-31-5p [170]. Low-intensity pulsed ultrasound (LIPUS) stimulation increased the secretion of exosomes from stem cells from the apical papilla (SCAP) without causing significant cytotoxicity or apoptosis. Exosomes from LIPUS-induced SCAP showed stronger efficacy in promoting osteogenic differentiation and reducing inflammation in periodontal ligament cells in vitro. In a mouse model of periodontitis, exosomes from LIPUS-induced SCAP effectively alleviated inflammation-induced bone loss. The study identified miRNA-935 as an important mediator of the pro-osteogenic and anti-inflammatory capabilities of LIPUS-induced SCAP exosomes [171]. Exosomes originating from TNF-α-preconditioned gingival mesenchymal stem cells (GMSCs) are further proven to be a potent regulator of the inflammatory process, specifically osteoclasts differentiation, which, in turn, might affect the course and treatment outcomes of periodontitis in a mouse model. TNF-α enhanced the exosomal miRNA-1260b expression that targets the Wnt5a-mediated RANKL pathway [172]. GMSC-Exos were reported also to significantly reduce the inflammatory response in PDLSCs induced by lipopolysaccharides (LPS). This was evidenced by decreased levels of pro-inflammatory cytokines (TNF-α and IL-1β) and increased levels of the anti-inflammatory cytokine IL-10. The anti-inflammatory effects of GMSC-Exos were mediated through the inhibition of the nuclear factor kappa B signaling pathway and the downregulation of Wnt5a expression [173].

Similarly, exosomes derived from human exfoliated deciduous teeth stem cells (SHED-Exos) reestablished bone levels in mouse periodontitis models and encouraged BM-MSC differentiation into bone-forming cells to initiate osteogenesis [174]. In addition, SHED-Exos were shown to participate in periodontal alveolar bone rejuvenation via different mechanisms, including neovascularization enhancement and bone deposition, probably using the adenosine monophosphate (AMP)-activated protein kinase signaling pathway [175]. The therapeutic potential of exosomes in periodontal diseases is summarized in Table 1. 

#### 3.3.2. Exosomes and Oral Cancer

Exosomal cargo has been documented to impact the tumor microenvironment, facilitating the development of tumors, immune resistance, metastasis, angiogenesis, and resistance to therapy [176]. When exosomes are employed in oral cancer therapy, the challenge is determining how to enhance the exosomes’ carrying capacity of the advantageous elements, particularly miRNAs. Exosomes loaded with cholesterol-modified miRNA-34a from human embryonic kidney 293T cells effectively inhibited proliferation, migration, and invasion of HN6 cells by downregulating special AT-rich sequence-binding protein 2 expression [177]. Exosomes derived from MSCs transfected with cabazitaxel (CTX) and TRAIL (MSCT-Exos/CTX) blocked the activation of phosphoinositide 3-kinase (PI3K), protein kinase B (Akt), and mTOR, potentially synergizing with CTX. Furthermore, MSCT-Exos and CTX induced apoptosis of SCC25 tumor cells [178]. Umbilical cord mesenchymal stem cell exosomes (UC-MSC-Exos) decreased the concentrations of inflammatory cytokines (IL-6 and TNF-α) and triggered apoptosis of oral squamous cell carcinoma (OSCC) in vitro [179]. SHED-Exos hindered cell growth; migration stimulated apoptosis and hampered the formation of tube-like structures in human umbilical vein endothelial cells (HUVECs) by decreasing various angiogenesis factors. This also reduced tumor-related blood vessel formation in xenografted OSCC cell tumors, resulting in tumor growth suppression in vivo [180].

##### Exosomes as Drug Delivery Vehicles for Oral Cancer

The primary therapeutic strategy for treating oral cancer is a combination of surgical removal of lesions and radiation therapy. However, the results of this treatment technique are not entirely satisfactory. Presently, the pursuit of new treatment approaches to minimize anti-cancer drug tolerance, enhance drug targeting, and raise the survival of patients is still a priority of research work. Exosomes have lately gained much interest as drug delivery vehicles as well as potential targets utilized against oral carcinomas [181]. The latest innovations in drug delivery techniques seek to improve targeting approaches, increase drug delivery rates, regulate drug release, and extend therapeutic activity. Exosomes have advanced drug delivery characteristics in comparison to liposomes, such as improved stability, which allows them to reach remote targeted tissues; a hydrophilic core that can incorporate water-soluble active ingredients; and greater biosafety, that do not evoke an immune reaction within the body [182,183]. Furthermore, through merging with the cytoplasmic membrane, attachment to specific surface membrane domains, and endocytosis, exosomes could smoothly deliver their cargo to target cells [184].

In an in vitro study, purified exosomes from BM-MSCs were electroporated with Dox (or anthracycline, which inhibits cancer cell growth by blocking topo-isomerase enzyme) to produce exosome-loaded doxorubicin (Exos-Dox). The efficacy of Exos-Dox was analyzed by Cell Counting Kit-8, and the adsorption and internalization of the drug was evaluated through inverted fluorescence microscopy and flow cytometry. After doxorubicin loading, the Exos-Dox diameter was relatively larger than that of free exosomes. When set side by side with free Dox, the assembled Exos-Dox demonstrated improved cellular uptake capability and a stronger anti-tumor influence in the osteosarcoma MG63 cell line [185]. See Table 2 for the therapeutic effect of exosomes in oral cancer.

#### 3.3.3. Exosomes and Sjögren’s Syndrome

Sjögren’s syndrome (SS) is a persistent autoimmune disorder affecting both the salivary and lacrimal glands, caused by lymphoid cell infiltrates that damage different tissues and negatively impact patients’ quality of life. Traditional SS diagnosis and treatment strategies are successful in alleviating symptoms using immunosuppressive drugs. However, long-term use of immunosuppressive drugs could result in a variety of systemic complications [186].

As shown in Table 3, exosomes have a therapeutic effect in Sjögren’s syndrome. MSC-derived exosomes could mimic MSCs’ immune modulation and, in turn, restore tissue homeostasis in immune-mediated conditions such as SS [187]. A study suggested that analogs of chromosome 19 miRNA cluster (C19MC)-derived miRNA present within exosomes were found to directly target immune cells both locally and systemically via conveying their load to targeted cells. The transferred miRNA could interact with the recipient cells’ mRNA within these target cells, inhibiting their specific immunity capabilities. As a result, this concept might be used in clinical trials for autoimmune diseases like SS to alleviate oral and ocular symptoms [188].

In experimentally induced SS (ESS), systemic administration of MSC-Exos significantly improved the saliva flow rate and reduced tissue damage in the salivary glands of ESS mice, in which olfactory ecto-mesenchymal stem cell-derived exosome treatment promoted myeloid-derived suppressor cell (MDSC) expansion with enhanced immunosuppressive function. This might represent a novel approach when treating SS, as well as other autoimmune conditions [189].

The EVs derived from early-passage human induced pluripotent stem cell-derived mesenchymal stem cells (iMSC-EVs) demonstrated superior immunomodulatory efficacy compared to late-passage iMSC-EVs, in both in vitro and in vivo models of SS [190]. Comparative molecular profiling revealed distinct molecular signatures, including TGF-β1, miRNA-21, and miRNA-125b, correlated with immunomodulatory potency. Modulation of these factors significantly impacted the immunosuppressive effects of iMSC-EVs. These findings provide valuable insights into the molecular mechanisms underlying iMSC-EV-mediated immunomodulation, offering potential avenues for optimizing EV-based therapies for autoimmune diseases [190]. Additionally, the therapeutic efficacy of lip gland mesenchymal stem cells exosomes (LGMSC-Exos) in treating SS was investigated in a mouse model for SS. LGMSC-Exos decreased inflammation, enhanced salivary gland functionality, and reorientated the immunological equilibrium towards a Treg phenotype. In vitro tests validated these findings, showing that LGMSC-Exos inhibited Th17 cell development and facilitated Treg cell production in peripheral blood mononuclear cells from SS patients. The findings indicate that LGMSC-Exos may serve as viable treatment alternatives for SS, providing both cellular and acellular techniques to regulate the immune response and rehabilitate salivary gland function [191].

DPSC-Exos were found to protect salivary gland epithelial cells from damage induced by interferon-gamma, a key inflammatory cytokine in SS. Mechanistically, DPSC-Exos activated the G protein-coupled estrogen receptor (GPER) signaling pathway, leading to increased cyclic AMP levels and subsequent activation of protein kinase A and cyclic AMP-response element-binding protein. This pathway upregulated aquaporin 5 expression, a crucial protein for salivary secretion, and restored salivary gland function. In vivo investigation in a mouse model of SS further confirmed the therapeutic efficacy of DPSC-Exos, demonstrating reduced inflammation and improved salivary flow. These findings suggest that DPSC-Exos may offer a novel therapeutic strategy for SS by targeting the GPER signaling pathway [192]. Moreover, SHED-Exos were applied in a mouse SS model. The study demonstrated enhanced saliva production in treated mice, suggesting improved functionality of the salivary glands. The SHED-Exos were internalized by glandular epithelial cells, increasing paracellular permeability through the upregulation of zonula occluden-1 (ZO-1) expression. The PI3K/Akt signaling pathway mediated this effect by influencing the expression of Slug, a transcription factor that negatively regulates ZO-1. The results indicate that the local application of SHED-Exos may be an effective therapeutic approach for mitigating hyposalivation in patients with SS [193]. Furthermore, UC-MSC-Exos demonstrated a potent immunomodulatory effect. UC-MSC-Exos effectively inhibited T cell proliferation, apoptosis, and Th17 differentiation, while simultaneously promoting Treg differentiation and reducing autophagy. These results highlight the therapeutic potential of UC-MSC-Exos in modulating the immune response and mitigating disease progression in SS [194].

**Table 3:** Therapeutic effect of exosomes in Sjögren’s syndrome.

Author/Year	Study Model		Source of Exosomes	Biological Activity	Outcome
	In Vitro	In Vivo			
Hai et al., 2018 [187,195]		Mouse model of SS.	- BM-MSCs - iPSC-MSCs	- Decreased lymphocyte infiltration in salivary glands. - Decreased serum autoantibody levels.	EVs from BM-MSCs and iPSC-MSCs had immunosuppressive properties and prevented the progression of SS before the onset of sialadenitis.
Rui et al., 2021 [189]		ESS mouse model.	Murine OE-MSC	- Improved salivary flow rate. - Decreased autoantibodies levels. - Reduced pathological changes in the submandibular glands. - Modulated the function of MDSCs with an immature phenotype and increased the expression of inhibitory factors.	OE-MSC-Exos had therapeutic potential to attenuate ESS progression by enhancing the immunosuppressive function of MDSCs.
Kim et al., 2021 [190]	PBMCs- SGECs coculture	Mouse model of SS.	iMSC	Modulation of key molecules, including TGF-β1, miRNA-21, and miRNA-125b, within iMSC-derived exosomes.	Early-passage iMSC-EVs demonstrated superior immunomodulatory efficacy compared to late-passage iMSC-EVs, both in vitro and in vivo models of SS.
Li et al., 2021 [191]	PBMCs from SS patients	Mice model of SS.	LGMSC	- Inhibition of T helper 17 cell development and facilitated Treg cell production in vitro. - Enhanced salivary gland functionality and reorientated the immunological equilibrium towards a regulatory T cell phenotype in vivo.	LGMSC-Exos may serve as a viable treatment alternative for SS.
Hu et al., 2023 [192]	SGEC treated with IFN-γ	Mice model of SS.	DPSCs	- In vitro, DPSC-Exos activated the GPER signaling pathway, leading to increased cAMP levels and subsequent activation of PKA and CREB. This pathway upregulated aquaporin 5 expression. - In vivo, DPSC-Exos demonstrated reduced inflammation and improved salivary flow.	DPSC-Exos enhanced the functionality of salivary gland epithelial cells in SS through the GPER-mediated cAMP/PKA/CREB pathway.
Du et al., 2023 [193]		Mice model of SS.	SHED	- Downregulated phospho-Akt (p-Akt)/Akt, phospho-glycogen synthase kinase 3b (p-GSK-3b)/GSK-3b, and Slug expressions and upregulated ZO-1 expression in submandibular glands	SHED-Exos improved SS-induced hyposalivation by improving glandular epithelial cell permeability via Akt/GSK-3b/Slug pathway-mediated ZO-1 expression.
Ma et al., 2023 [194]	SS patients CD4^+^ T cells		UCMSC	- Inhibition of T cell proliferation, apoptosis, and Th17 differentiation, - Promoted Treg differentiation and reduced autophagy.	UCMSC-Exos exerted potent immunomodulatory effects on CD4^+^ T cells.

BM-MSCs: Bone marrow mesenchymal stem cells; cAMP: cyclic adenosine monophosphate; CREB: G protein-coupled estrogen receptor; DPSCs: Dental pulp mesenchymal stem cells; ESS: Experimentally-induced Sjögren’s syndrome; EVs: Extracellular vesicles; iMSC: Human induced pluripotent stem cell-derived mesenchymal stem cells; Exos: Exosomes; IFN-γ: Interferon gamma; iPSC: Induced pluripotent stem cells; LGMSC: Labial gland-derived MSCs; MDSCs: Myeloid-derived suppressor cells; miRNA: MicroRNA; MVs: Microvesicles; NO: Nitric oxide; OE-MSC: Olfactory ectomesenchymal stem cells; PBMCs; Peripheral blood mononuclear cells; PKA: Protein kinase A; ROS: Reactive oxygen species; SGECs: Salivary gland epithelial cells; SHED: Stem cells derived from human exfoliated deciduous teeth; SS: Sjögren’s syndrome; UC-MSC: Human umbilical cord mesenchymal stem cells.

#### 3.3.4. Exosomes and Craniofacial Bone Loss

Autogenous and allogeneic bone grafts have been extensively used to treat bone defects. However, there are still several limitations regarding using these bone grafts [196]. Alternatively, the angiogenic and osteogenic potentials of MSC-derived exosomes have been reported to be applied in cell-free therapy to treat bone loss [197]. For example, dimethyloxaloylglycine-stimulated human BM-MSC-derived exosomes triggered the Akt/mTOR pathway for angiogenesis stimulation in HUVECs in vitro [198]. BM-MSC-derived exosomes were also reported to enhance proliferation, osteoblastic differentiation, and alkaline phosphatase (ALP) activity of human osteoblasts (hFOB1.19 cells) in vitro via miRNA-21-5p mediated inhibition of Kruppel-like factor 3 [199]. Furthermore, BM-MSC-derived exosomes promoted type H blood vessel angiogenesis coupled with osteogenesis to accelerate bone regeneration in an osteoporosis mouse model. Exosomal miRNA-150-5p targets SRY-box transcription factor 2 was suggested to be the key mechanism mediating angiogenesis by regulating oxidative phosphorylation and the PI3k/Akt signaling pathway in the osteogenic microenvironment [200]. Another work by Cao et al. demonstrated that miRNA-21-5p in ADSC-derived exosomes promoted angiogenesis and bone repair by regulating the neurogenic locus notch homolog protein 1/delta-like protein 4/vascular endothelial growth factor (VEGFA) signaling pathway [201].

It has been reported that SHED-Exos enhance the process of osteogenic differentiation in PDLSCs. This is evidenced by the presence of intense Alizarin red staining, increased ALP activity, and increased expression of osteogenic genes such as Runt-related transcription factor 2 (RUNX2), Osteopontin, and Osteocalcin (OCN) [202]. In another work, exosomes derived from SHEDs combined with DPSCs was found to promote osteogenic differentiation of DPSCs in vitro and enhance cranial and mandibular bone regeneration in vivo. By transferring mitochondrial transcription factor A (TFAM) mRNA, and increasing TFAM expression, exosomes mechanistically activated mitochondrial oxidative phosphorylation activity and triggered osteogenic differentiation in DPSCs [203]. Loading of exosomes on scaffolds has also been reported to improve the osteogenic potential of exosomes. For example, human endometrial mesenchymal stem stromal cell-derived exosomes loaded on hydroxyapatite scaffolds enhanced angiogenesis and osteogenesis in rat calvarial defects, as confirmed by hematoxylin-eosin staining, Masson’s trichrome staining, immunohistochemistry, and histomorphometric analysis [204]. Moreover, exosomes derived from osteogenically committed human UC-MSCs, when incorporated in 3D printed tricalcium phosphate scaffolds, significantly accelerated new bone formation in cranial bone defects in a rat model [205]. Similary, rats that were locally treated with DPSC-Exos laden with collagen membrane exhibited increased new bone density at the mandible defects [206]. Table 4 highlights the therapeutic effect of exosomes in craniofacial bone loss.

#### 3.3.5. Exosomes and TMJ Diseases

MSCs exosomes were reported to have the ability to induce cartilage repair and regeneration [144]. Zhang et al. assessed the effects of human embryonic stem cell (ESC)-derived MSC exosomes’ intra-articular injections in a monosodium iodoacetate-induced TMJ-osteoarthritis rat model (TMJOA). It was demonstrated that MSC exosomes alleviated the degenerated TMJ in an immunocompetent rat model by repressing inflammation and pain, inhibiting fibrosis, besides stimulating TMJ matrix restoration of the condylar cartilage and subchondral bone after eight weeks post-treatment [195]. Similarly, BM-MSC-small EVs were observed to significantly upregulate the expression of proliferating cell nuclear antigen and cartilage-forming factors and downregulate expression of cartilage inflammation-related factors in a rabbit TMJOA model. The authors showed that BM-MSC-small EVs promoted cartilage reconstruction via the autotaxin-Yes-associated protein signaling axis [207].

miRNA-100-5p-carrying SHED-Exos were reported to suppress the inflammation in TMJ chondrocytes with the downregulation of pro-inflammatory enzyme expression through inhibition of mTOR signaling pathways [208]. In another study by Li et al., ADSCs-Exos showed greater bioactivity in promoting the migration, proliferation, and chondrogenic and osteogenic differentiation of BM-MSCs in vitro, as well as cartilage and bone regeneration in a xenograft mouse model when compared to exosomes of MSCs derived from bone marrow and synovium. The exosomes’ cartilage and bone regenerative capacities were suggested to be attributed to the different mechanisms associated with the focal adhesion, ECM-receptor interaction, actin cytoskeleton, cyclic AMP, and PI3K/Akt signaling pathways [209].

Additionally, it was reported that inflammation-stimulated ADSC-derived small EVs (IAE) and normal ADSC-derived small EVs promoted TMJ regeneration in the osteochondral condylar defect in rabbits’ TMJ after 8 weeks [210]. The IAE group had the best new subchondral bone quality, increased bone volume, trabeculae, and trabecular bone thickness, with less trabecular separation [210]. Table 5 demonstrates the therapeutic effect of exosomes in TMJ diseases.

#### 3.3.6. Exosomes and Dentin Pulp Complex Regeneration

The angiogenic role of SHED aggregate-derived exosomes (SA-Exos) was explored in pulp regeneration. SA-Exos were enriched by miRNA-26a, which promoted angiogenesis of SHEDs and HUVECs via the TGF-β/SMAD2/3 signaling pathway, where angiogenesis-associated gene expression levels of VEGF, angiogenin, and platelet-derived growth factor were significantly upregulated after SA-Exos treatment [211]. Further, SA-Exos loaded on tooth fragments and implanted subcutaneously in immune-deficient mice showed promising results in dentin–pulp complex regeneration in vivo [211]. HUVECs were treated by hypoxic-preconditioned SHED exosomes (Hypo-Exos), to mimic the physiological root resorption of deciduous teeth, and exosomes from routinely cultured SHEDs (Norm-Exos). HUVECs’ growth, migration, and tube formation were substantially improved by Hypo-Exos in vitro when contrasted with Norm-Exos. Hypo-Exos transported both let-7f-5p and miRNA-210-3p, which enhanced the creation of tubes by endothelial cells, suggesting that these two exosomal miRNAs have a role in regulating angiogenesis. Hypo-Exos also showed improved angiogenesis in vivo upon loading on Matrigel followed by subcutaneous implantation in nude mice [212].

DPSC-derived exosomes improved human BM-MSC proliferation and migration in combination with fibrin gel, which was used as a delivery system of exosomes, proposing the role of injectable fibrin hydrogel as an effective tool for cell-free regenerative endodontics via chemotaxis of MSCs [213].

DPSC-Exos have been shown to improve the odontogenic differentiation of DPSCs by means of their exosomal miRNA. The protein expressions of dentin sialophosphoprotein (DSPP), dentin matrix protein (DMP)1, ALP, and RUNX2 were upregulated by miRNA-27a-5p mimics, which promoted odontogenic differentiation of DPSCs by activating the TGFβ1/SMADs signaling pathway. This was confirmed by an increase in TGFβ1, TGFR1, p-SMAD2/3, and SMAD4 proteins, as evidenced by the Western blot results [214]. Human DPSCs-Exso, especially from LPS preconditioned human DPSCs, significantly promoted the proliferation, migration, and odontogenic differentiation of Schwann cells. The mRNA levels of odontogenic differentiation marker genes DSPP, DMP1, OCN, and RUNX2 were upregulated after exosome treatment. Furthermore, the formation of reddish-brown mineralized nodules was observed by Alizarin Red staining, indicating increased mineralization capacity [215].

Moreover, the odontogenic potential of SCAP-Exos was investigated in vivo. SCAP-Exos was introduced into the root fragment containing BM-MSCs and transplanted subcutaneously into immunodeficient mice. After 12 weeks of treatment with SCAP-Exos, histological analysis showed a formation of a new continuous dentine layer with a significant increase in the number of polarized columnar-shaped odontoblasts located at the predentine pulp junction [216].

Shi et al. demonstrated a potent effect of exosomes derived from human ESC-derived MSCs on dentin pulp complex regeneration, with a significant upregulation of viability, proliferation, and migration of DPSCs associated with upregulation of fibroblast growth factor-2, IGF-1 and Ki67 genes and reduced cellular apoptosis of DPSCs via suppression of Bax and upregulation of Bcl-2. Human ESC-derived exosomes further enhanced odontogenic differentiation and calcium deposition of DPSCs, along with upregulation of markers such as DSPP and DMP1, matrix extracellular phosphoglycoprotein, bone morphogenetic protein2 (BMP2), OCN, TGF-β1, ALP, BMP7, and bone sialoproteins. Through the same study, formation of a dentin bridge was recorded after implantation of ESC-derived exosomes loaded on a collagen sponge in a rat model of pulpal defect. In another immunodeficient mouse model, pulpal tissue regeneration occurred within empty root canals of premolars that were injected with ESC-derived exosomes loaded on collagen and implanted subcutaneously [217].

Exosomes extracted from LPS-stimulated human DPSCs to mimic inflammatory conditions was investigated for its dentin pulp complex regenerative potential. LPS-stimulated human DPSC-derived exosomes significantly stimulated the angiogenic potential of HUVECs with significant upregulation of proliferation, migration, and tube formation of HUVECs in vitro in addition to significant upregulation of VEGF and kinase-insert domain-containing receptor, as compared to non-stimulated human DPSCs, highlighting the role of inflammation in potentiating the angiogenic effect of human DPSCs with potential application in dental pulp revascularization and regeneration [218].

Similarly, small EVs originating from LPS-preconditioned human DPSCs effectively upregulated BM-MSC proliferation and angiogenesis using and ECM-2 Bullet Kit and seeding upon Matrigel in vitro, and was associated with upregulation in the expression of ALP, DSPP, Neurofascin, and VEGF. While in vivo, small EVs injected into empty, pulp-less, root canals of rats showed pulpal regeneration, in addition to mineralized tissues formation when combined with BM-MSCs [219].

Moreover, exosomes derived from dental pulp tissues and DPSCs of swine significantly promoted SCAP and HUVEC proliferation and migration, and enhanced the angiogenic effect and tube formation of HUVECs seeded on Matrigel. Additionally, exosomes derived from dental pulp tissues enhanced SCAP odontogenic potential, increased ALP levels, and mineralized nodule formation. Both types of exosomes were loaded on collagen gel, loaded on treated dentin matrix, and implanted subcutaneously in nude mice. They successfully regenerated and vascularized pulp-like tissues associated with the formation of predentin-like tissue, lined with odontoblast-like cells. It was concluded that exosomes can recruit tissue resident SCAP to aid in dentin pulp complex regeneration [220]. Table 6 outlines the therapeutic effect of exosomes in dentin pulp complex regeneration.

## 4. Exosomes as a Diagnostic Biological Marker

Exosomes are considered a non-invasive biomarker for the diagnosis of several diseases. They are found expansively in various bodily fluids. Exosomes have higher sensitivity and specificity than standard biomarker specimens such as serum or urine due to their superior stability [221]. Exosomes have a specific molecular content that identifies their parental cell type. Their component levels depend mainly on the cells’ functional status, whether the cells are in a normal physiological or pathological state. Consequently, exosomal cargo analysis manifests the altered parental cell state and offers visions for various disease diagnoses [222]. Exosomes are easy to analyze and can be kept for later use at −80 °C for a week to two years, depending on the source of the exosomes [223].

### 4.1. Diagnosis of the Aging Processes

The aging effect was investigated on serum EVs, where CD63 levels and acetylcholinesterase (AChE) activity were analyzed as markers of exosomes. Reduction in CD63 levels and increased AChE activity and reactive species in circulating exosomes were reported in aged rats compared to young ones [224]. Moreover, Eitan et al. analyzed circulating plasma EVs in a cross-sectional and longitudinal study aiming to highlight age-related changes in community-dwelling individuals within five years. EV concentration was reported to decline with aging [225]. 

### 4.2. Diagnosis of Age-Related Oral Diseases

#### 4.2.1. Periodontal Diseases

Genetically, exosomes are supplemented with specific miRNAs that can offer disease-specific diagnostic remarks. Periodontitis patients’ plasma-derived exosomal miRNA (miRNA-1304-3p and miRNA-200c-3p) and small nucleolar RNA signatures (SNORD57 and SNODB1771) are differentially expressed when compared to healthy controls, suggesting that they could be useful biomarkers for periodontitis diagnosis [226].

By investigating and characterizing salivary exosomal proteins in young adults with severe periodontitis, it is suggested that complementary component 6 (C6) proteins, which participate in the immune response during periodontitis development, were expressed only in the severe periodontitis group [183]. Furthermore, CD9 and CD81 marker levels in periodontitis patients were dramatically inferior to healthy controls. Consequently, it was concluded that salivary markers such as CD9 and CD81 could be significantly and inversely related to clinical periodontal measurements, thereby important in the diagnosis and prognosis of periodontal disease [227].

Additionally, the programmed death-ligand 1 (PD-L1) mRNA level was assessed in salivary exosomes from periodontitis patients and non-periodontitis controls. The level of PD-L1 mRNA in salivary exosomes was highly elevated in periodontitis. Its level is linked with the severity of periodontitis, which suggests that salivary exosomal PD-L1 mRNA could be a practicable periodontitis biomarker [228].

#### 4.2.2. Oral Cancer

Surgical tissue biopsy is currently regarded as the gold standard for solid tumor diagnosis. However, this morphological-dependent procedure is physically invasive and time-consuming, and might cause undue pain and anxiety for the patient. Furthermore, different sites of the primary tumor display substantial inter- and intra-tumor variations, making it challenging to achieve an accurate and successful treatment protocol based on just one biopsy, necessitating other invasive tissue samples [229].

In this context, exosome-based liquid biopsies have gained a great deal of interest from physicians and researchers over the last few years because of their ease of access, comfort, non-invasiveness, time and effort conservation, reliable validity and reproducibility, ease of early identification, and relatively inexpensiveness, together with high efficiency [230]. Apolipoprotein A1, platelet factor 4 variant 1, C-X-C motif chemokine ligand 7, and coagulation factor XIII in serum-derived exosomes were shown to be advantageous as diagnostic biomarkers for identifying OSCC spread in distant parts of the body. Pairing these biological markers could optimize OSCC diagnosis acuity and subsequently decrease misdiagnosis in patients suffering from lymph node metastasis [231]. Similarly, a study compared the miRNA microarray analysis of salivary exosomes from patients with OSCC to the control salivary specimens to determine the differences in miRNA expression profiles.Researchers discovered a substantial rise in the amount and irregular morphological characteristics of salivary exosomes in oral cancer. These findings indicate that the shape, number, surface biomarkers, and composition of saliva-derived exosomes differ between oral cancer patients and healthy controls, and that exosomes might be the “cornerstone” to early diagnosis of oral cancer [232].

#### 4.2.3. Salivary Gland Diseases

An initial experiment to yield exosomal miRNA from SS patients’ parotid saliva was carried out by Michael et al., hypothesizing that the miRNA load of salivary exosomes might afford biomarkers for the diagnosis of numerous salivary gland conditions, including SS [233]. The screening of salivary and tear specimens from SS subjects using liquid chromatography coupled with mass spectrometry aided in identifying potential innovative biomarkers involving oral and eye diseases. Salivary exosomes also exhibited biomarkers such as signal regulatory protein alpha and lymphocyte-specific protein 1, crucial for triggering non-specific immune reactions. This novel strategy might improve diagnostic preciseness in SS, and also could be used for disease staging and monitoring [234].

A more recent study revealed that T cell-derived exosomes incorporating miRNA-142-3p could trigger SS pathogenesis. When miRNA-142-3p-loaded exosomes are delivered inside salivary gland epithelial cells, they could impair cytoplasmic Ca^2+^ signaling, reduce cyclic AMP generation, and decrease the production of proteins from parenchymal gland cells, resulting in impairment of glandular cells [235].

Despite these encouraging results, there is a lack of comprehensive findings supporting the application of exosomes or exosomal miRNA as valid biomarkers of SS. As a result, additional investigations are mandatory to verify the essential implications of exosomes or exosomal miRNA as reliable, precise, and sensitive biological markers for SS [236].

### 4.3. Translation of Exosome-Based Diagnostic and Treatment Challenges

Although exosomes hold great potential for diagnosing and treating a wide range of diseases, there are still obstacles to moving this technology from the lab to the clinic. The primary challenge in the therapeutic use of exosomes is the need for dependable, standardized, and specific methods for isolating them. Several approaches for exosome separation have been used, including ultracentrifugation, polymer precipitation, and ultrafiltration-based approaches; however, they all have several disadvantages, making them challenging to apply to large-scale downstream clinical purposes [237]. Additionally, exosomes are frequently difficult to separate and detect in body fluids at a low cost while maintaining acceptable specificity and sensitivity due to their small size and sample variability. As a result, establishing a reliable and consistent method to collect exosomes for therapeutic and diagnostic applications is critical [237].

Major limitations in isolation of exosomes are contamination with non-exosomal vesicles, co-isolation of protein aggregates and lipoproteins, and damage to vesicle membranes, in addition to the need for specialized equipment and lengthy preparation time [238]. To address these limits, researchers have adopted creative approaches, such as combining two or more separation techniques and utilizing microfluidics-based methods to achieve exact isolation with less contamination of other cell components [237]. Microfluidics is excellent for clinical usage due to its low cost, requirement for less experimental time, and high sensitivity for detection, particularly in the field of disease diagnosis and prognosis by examining miRNAs present in body fluids [239]. Even though microfluidic technologies are frequently touted as a “cost-effective” alternative to traditional benchtop-scale instruments for exosome isolation when compared to other conventional approaches in use, this classification remains arbitrary because it is difficult to compile accurate cost data for various microfluidic systems [237]. When it comes to thorough clinical testing and validation in patient cohorts, microfluid systems are presented as being limited to lab research settings, and adequate procedure standardization is still being investigated to ensure system reproducibility [240,241,242].

Lack of standardization in characterization, purification, stabilization, enrichment, storage, and source of MSCs from which exosomes are derived, and culture systems for MSCs, are considered obstacles facing exosome-based therapy [243]. These issues lead to heterogeneity of exosomes regarding their size, internal biological components, impact on the recipient cells’ functionality, membrane markers, and proteomic characteristics [244].

Another challenge limiting the applicability of exosomes is concerned with their scalability and poor yield. MSCs, the most common source of exosomes used, have a limited capacity to produce exosomes to exert beneficial clinical effects [245]. Researchers focus on different approaches for enhancing exosome large-scale production where two primary strategies are employed: the first involves genetically modifying the exosome biogenesis and release route; the other involves preconditioning of mother cells, altering the culture technique, or adding distinct elements into the medium. Applying these techniques would enable the mass production of exosomes, which in turn boosts their effectiveness in clinical applications [246].

Exosome-based therapies may also suffer from exosome off-targeting, resulting in low therapeutic potential. As the use of the natural targeting mechanisms of exosomes alone is insufficient for accurate targeting, optimization of natural targeting and delivery methods could be more effective in clinical approaches [232].

Finally, upon clinical applications, more questions arose regarding the delivery pathway, timespan needed to treat the disease, number of doses, safety, and potency [247,248].

## 5. Conclusions

Aging is an unavoidable pathophysiological process caused by several molecular processes and pathways that contribute to cellular, tissue, and organismal declines in function. Various soft and hard oral tissue diseases had been reported to be associated with the aging process. As we have exposed in this review, exosomes derived from different MSC sources demonstrated anti-oxidant, anti-inflammtory, tissue regeneration, and immunomodulatory effects in both in vitro and in vivo models of several oral and para-oral disorders that are more common with aging, such as periodontal diseases, oral cancer, salivary gland diseases, and TMJ disorders. Moreover, the importance of exosomes as valid biomarkers in different age-related oral diseases was pointed out in this article. Therefore, MSC-derived exosomes could offer a promising strategy for diagnosing and treating various age-related oral diseases.

Yet, translating exosome-based therapy from preclinical studies to clinical trials still faces many challenges. Further studies are needed to improve exosomal isolation, explore the impact of exosomes on the functionality of recipient cells, and address the legal, ethical, and regulatory concerns.

## Figures and Tables

**Table 1:** Therapeutic effect of exosomes in periodontal diseases.

Author/Year	Study Model		Source of Exosomes	Biological Activity	Outcome
	In Vitro	In Vivo			
Shen et al., 2020 [165]		Periodontitis mouse model.	DPSCs	- Immunomodulatory effects of DPSC-Exos/CS are associated with miRNA-1246 in DPSC-Exos.	DPSC-Exos promoted amelioration of periodontitis.
Qiao et al., 2023 [166]	- PDLSCs - RAW264.7 cells	Periodontitis rat model.	DPSCs	- Enhanced expression of Col1a1, DSPP, OCN, and OPN genes as well as ALP, IL-10 and TGF-β. - Downregulated IL-6, TNF-α, p-JAK2, and p-STAT3.	DPSC-Exos modulated key genes and proteins involved in inflammation and immune responses, thereby ameliorating periodontitis.
Chen et al., 2024 [167]	Human PDLCs	Periodontitis rat model.	BM-MSCs	- Enhanced OPN, OCN, Col1, and FN. - Downregulation of RANKL and upregulation of OPG and TGF-β1. - Downregulation of iNOS and CD86, while upregulation of Arg-1 and CD163 in treated macrophages.	BM-MSC-small EVs promoted alveolar bone and periodontal ligament repair, reducing alveolar bone loss, and inflammation.
Yue et al., 2022 [168]	RAW 264.7	Periodontitis rat model.	BM-MSCs	- Reduction of pro-inflammatory cytokines IL-6, IL-1β, and TNF-α, MMP-9, Ccl7, and Lst1. - Enriched anti-inflammatory miRNA-100-5p, miRNA-125b-5p, and miRNA-21-5p.	BM-MSC-Exos managed periodontitis by modulating immune responses and reducing inflammation.
Shimizu et al., 2022 [169]	- HHH-DPCs - Mouse osteoblastic MC3T3-E1 cells	Periodontitis mouse model.	HLA-HHH treated DPCs	- Suppression of RANKL and osteoclast formation.	HHH-DPC-Exos attenuated bone loss, reversing periodontitis.
Lu et al., 2023 [170]	RAW264.7	Diabetic periodontitis mouse model.	Normal-glucose- and high-glucose-PDLSCs	- Downregulation of TRAF6, NFATc1, c-Fos. - Upregulation of miRNA-31-5p. - eNOS downregulated by miRNA-31-5p.	NG-PDLSC-Exos inhibited osteoclast formation and differentiation more than HG-PDLSC-Exos.
Zhang et al., 2023 [171]	Human PDLCs	Periodontitis mouse model.	SCAP	- Downregulation of TNF-α, IL-1β, IL-6, and Wnt5a. - Upregulation of IL-10 and nSMase2.	LIPUS enhanced production and therapeutic efficacy of SCAP-Exos for treating inflammation-induced bone loss.
Nakao et al., 2021 [172]	PBMC-derived macrophages	Periodontitis mouse model.	TNF-α-treated human GMSCs	- Anti-inflammatory M2 macrophage polarization. - Anti-osteoclastogenic activity.	TNF-α-treated GMSC-Exos reduced periodontal bone resorption.
Sun et al., 2022 [173]	PDLSCs		GMSCs	- Decreased levels of TNF-α, Wnt5a and IL-1β - Increased levels of IL-10. - Inhibition of NF-κB signaling pathway.	GMSC-Exos attenuated the inflammatory response in PDLSCs and promoted periodontal tissue regeneration.
Wei et al., 2020 [174]	BM-MSCs	Periodontitis mouse model.	SHEDs	- Upregulation of p-Smad5 and RUNX2. - Decrease expression of the adipogenic marker PPARγ and the amount of lipid droplets. - Inhibition of IL-6 and TNF-α.	SHED-Exos promoted BM-MSC osteogenesis, differentiation, and bone formation.
Wu et al., 2019 [175]	- HUVECs - BM-MSCs	Periodontal defect rat model.	SHEDs	- Upregulation of: angiogenic genes (KDR, SDF-1, FGF2), - Osteogenic genes (COL1, RUNX2, OPN)—p-AMPK.	SHED-Exos enhanced periodontal bone regeneration by promoting neovascularization and new bone formation.

ALP: Alkaline phosphatase; Arg-1: arginase 1; BM-MSCs: Bone marrow mesenchymal stromal cells; β-TCP: β-tricalcium phosphate; Ccl7: Chemokine (C-C motif) ligand; COL1: Type I collagen; CS: Chitosan; DPCs: Dental pulp cells; DPSCs: Dental pulp stem cells; DSPP: Dentin sialophosphoprotein; eNOS: Endothelial nitric oxide synthase; EV: Extracellular vesicles; Exos: Exosomes; FGF2: Fibroblast growth factor-2; FN: Fibronectin; GMSCs: Gingival mesenchymal stem cells; HG-PDLSCs: High-glucose-preconditioned PDLSCs; HLA HHH: Human leukocyte antigen haplotype homo; HUVECs: Human Umbilical Vein Endothelial Cells; IL: Interleukin; iNOS: Inducible Nitric Oxide Synthase; KDR: Kinase insert domain receptor; LIPUS: Low-intensity pulsed ultrasound; LST1: Leukocyte Specific Transcript 1; miRNA: Micro RNA; MMP-9: Matrix Metalloproteinase-9; NFATC1: Nuclear factor of activated T cells 1; NF-κB: Nuclear factor kappa B; NG-PDLSCs: Normal-glucose-cultured periodontal ligament stem cells; OCN: Osteocalcin; OPG: Osteoprotegerin; OPN: Osteopontin; p-AMPK: Phosphorylated activated protein kinase; PBMC: Peripheral blood mononuclear cells; PDLCs: Periodontal ligament cells; PDLSCs: Periodontal ligament stem cells; PPARγ: Peroxisome proliferator-activated receptor γ; RANKL: Receptor activator of nuclear factor kappa beta ligand; RUNX2: Runt-related transcription factor 2; SCAP: Stem cells from the apical papilla; SDF-1: Stromal cell–derived factor 1; SHEDs: Human exfoliated deciduous teeth; TGF-β1: Transforming growth factor beta 1; TNF-α: tumor necrosis factor alpha; TRAF6: Tumor necrosis factor receptor associated factor 6.

**Table 2:** Therapeutic effect of exosomes in oral cancer.

Author/Year	Study Model		Source of Exosomes	Biological Activity	Outcome
	In Vitro	In Vivo			
Deng et al., 2022 [177]	OSCC		HEK293T cells	miRNA-34a-loaded exosomes inhibited HN6-OSCC cell proliferation, migration, and invasion by down regulating SATB2 expression.	New delivery method for miRNA-34a to treat OSCC.
Qiu et al., 2020 [178]	OSCC		MSCT	CTX incorporated in MSCT-EXOs inhibit the activation of PI3K, Akt and mTOR, and induce the apoptosis of tumor cells in a dose-dependent manner.	MSCT-Exos-based CTX delivery system could be used treat OSCC.
Abdelwhab et al., 2023 [179]	OSCC		UC-MSCs-	- Reduction of pro-inflammatory cytokines levels. - Induction of apoptosis. - Downregulate HOTAIR.	UC-MSC-Exos conferred a suppressive role on OSCC.
Liu et al., 2023 [180]	HUVECs	- Chick chorioallantoic membrane assay. - Xenograft transplantation model of OSCC in nude mice.	SHED	- Downregulation of several angiogenesis-related factors. - Suppress micro-vascular formation.	SHED-Exos exhibited an antitumor effect through inhibiting angiogenesis.
Wei et al., 2019 [185]	- Human osteosarcoma cell line MG63		BM-MSCs	- Enhanced cellular uptake efficiency and anti-tumor effect.	BM-MSC-Exos-loaded Dox could serve as good candidate for targeted osteosarcoma treatment.

BM-MSCs: Bone marrow mesenchymal stem cells; CTX: Cabazitaxel; Dox: Doxorubicin; Exos: Exosomes; HEK: Human embryonic kidney; HOTAIR: HOX transcript antisense intergenic long noncoding RNA; HUVECs: Human umbilical vein endothelial cells; HEK: Human embryonic kidney; miRNA: Micro RNA; MSCs: Mesenchymal stem cells; MSCT: Mesenchymal stem cells-TRAIL; OSCC: Oral squamous cell carcinoma; SATB2: Special AT-rich sequence-binding protein2; SHED: Human deciduous exfoliated teeth stem cells; TRAIL: Tumor necrosis factor-related apoptosis-inducing ligand; UC-MSCs: Umbilical cord mesenchymal stem cells.

**Table 4:** Therapeutic effect of exosomes in craniofacial bone loss.

Author/Year	Study Model		Source of Exosomes	Biological Activity	Outcome
	In Vitro	In Vivo			
Liang et al., 2019 [198]	HUVECs	Calvarial defect rat model.	BM-MSCs	- Promoted tube formation in vitro. - Increased new vessels formation and increased new bone formation and mineralization in vivo.	DMOG-human BM-MSC-Exos enhanced neovascularization and bone regeneration.
You et al., 2022 [199]	Human FOB1.19 cells		BM-MSCs	Enhanced cell viability, differentiation, and ALP activity.	BM-MSC-Exos enhanced osteogenesis.
Wu et al., 2024 [200]	EPCs	Osteoporosis mice model.	BM-MSCs	- Promoted cell migration and proliferation. - Upregulated CD31 and Emcn protein and mRNA expression in vitro. - Increased immunoexpression of CD31 and Emcn. - Improved trabecular structure and attenuate the bone loss in vivo.	BM-MSC-Exos enhanced angiogensis and bone regeneration.
Cao et al., 2024 [201]	EPCs	Cranial defect rat model.	ADSCs	- Promoted cell viability, migration, invasion, and tube formation in vitro. - Upregulated the gene and protein expression levels of CD31, VEGFA, OCN, and RUNX2.	ADSCs-Exos promoted angiogenesis to accelerate bone regeneration.
Wang et al., 2022 [202]	Human PDLCs		SHED	- Increased cell migration, proliferation and osteogenic differentiation. - Increased RUNX2 expression.	SHED-Exos enhanced osteogenesis.
Guo et al., 2022 [203]	DPSCs	- Cranial defect mice model. - Mandibular defect rat mode.l	SHED	- Increased expression of osteogenic genes and proteins (ALP, RUNX2 and BMP2) in vitro. - Increased new bone formation and more collagen deposition in vivo.	SHED-Exos combined with DPSC-enhanced bone regeneration.
Youseflee et al., 2022 [204]		Calvarial defect rat model.	Human EnSCs	New bone tissue formation.	Human EnSCs-Exos enhanced osteogenesis and angiogenesis.
Li et al., 2024 [205]	BM-MSCs	Cranial defect rat model.	Human UC-MSCs	- Increased ALP activity, and upregulated RUNX2, COL1, and OCN gene expression in vitro. - Enhanced mature bone tissue formation in vivo.	Human UC-MSC-Exos promoted bone regeneration.
Lee et al., 2023 [206]	JB-MSCs	Mandibular defect rat model.	DPSCs	- Increased expression of (RUNX2, OCN) in vitro. - Increase new bone formation in vivo.	DPSCs-EVs induced an osteogenic and osteoinductive effects.

ADSCs: Adipose-derived stem cell; ALP: Alkaline phosphatase; BM-MSCs: Bone marrow mesenchymal stem cells; BMP2: Bone morphogenetic protein 2; CD31: Platelet/endothelial cell adhesion molecule; COL1: Type I collagen; DMOG-human BM-MSC: Dimethyloxaloylglycine-stimulated human bone marrow- mesenchymal stem cells; DPSCs: Dental pulp stem cells; Emcn: Endomucin; EnSCs: Endometrial mesenchymal stem stromal cells; EPCs: Endothelial progenitor cells; EVs: Extracellular vesicles; Exos: Exosomes; FOB1.19 cells: Human osteoblasts; HUVECs: Human umbilical vein endothelial cells; JB-MSCs: Jawbone marrow-derived mesenchymal stem cells; OCN: Osteocalcin; PDLCs: Periodontal ligament stem cells; RUNX2: Runt-related transcription factor 2; SHED: Human exfoliated deciduous teeth; UC-MSCs: Human umbilical cord mesenchymal stem cells; VEGFA: Vascular endothelial growth factor A.

**Table 5:** Therapeutic effect of exosomes in TMJ diseases.

Author/Year	Study Model		Source of Exosomes	Biological Activity	Outcome
	In Vitro	In Vivo			
Zhang et al., 2019 [195]		TMJOA rat model.	Human embryonic stem cells	- Enhanced s-GAG synthesis. - Suppressed IL-1β-induced nitric oxide and MMP13 production.	MSC-Exos promoted TMJ repair and regeneration.
Wang et al., 2021 [207]	MCCs	TMJOA rabbit model.	BM-MSCs	- Upregulated gene expression of PCNA, COL-II, ACAN, and SOX9. - Downregulated gene expression of RUNX2 and MMP13 in vitro. - Increased hyaline cartilage formation in vivo.	BM-MSC-small EVs promoted cartilage reconstruction.
Luo et al., 2019 [208]	Human chondrocytes		SHED	Suppressed expression of IL-6, IL-8, MMP1, MMP3, MMP9 and MMP13.	SHED-Exos suppressed inflammation in TMJ chondrocytes.
Li et al., 2021 [209]	BM-MSCs	Xenograft mouse model.	- ADSCs - BMSCs - SMSCs	- Upregulated the expressions of hyaline cartilage-specific genes (ACAN, COL-II, SOX9) and osteogenic genes (OCN, COL-I, RUNX2) in vitro. - Aggregation of chondrocytes into cartilage lacuna-like structures. Bone-like tissue formation, which was evidenced by the presence of haversian canals like structures in vivo.	- ADSC-small EVs, BMSC-small EVs, and SMSC-small EVs enhanced chondrogenesis and osteogenesis. - ADSCs-EVs showed the best bioactivity.
Liu et al., 2022 [210]	- BM-MSCs - Macrophages	TMJ condylar osteochondral defect rabbit model.	ADSCs	- Promoted BM-MSCs proliferation and migration as well as promoted M2 macrophage differentiation in vitro. - New subchondral bone & hyaline cartilage formation in vivo.	ADSC-small EVs had anti-inflammatory and pro-repair effect in TMJ leading to its regeneration and repair.

ACAN: Aggrecan; ADSCs: Adipose-derived stem cell; BM-MSCs: Bone marrow mesenchymal stem cells; Col-II: Collagen II; EVs: Extracelluar vesicles; Exos: Exosomes; IL-1β: Interleukin 1 beta; MCCs: Mandibular condylar chondrocytes; MMP: Matrix metalloproteinase; MSCs: Mesenchymal stem cells; OCN: Osteocalcin; PCNA: Proliferating cell nuclear antigen; RUNX2: Runt-related transcription factor 2; s-GAG: Sulfated glycosaminoglycans; SHED: Human exfoliated deciduous teeth; SMSCs: Synovium mesenchymal stem cells; SOX9: SRY-related high-mobility group box 9; TMJOA: Temporomandibular joint osteoarthritis.

**Table 6:** Therapeutic effect of exosomes in dentin pulp complex regeneration.

Author/Year	Study Model		Source of Exosomes	Biological Activity	Outcome
	In Vitro	In Vivo			
Wu et al., 2021 [211]	HUVECs	Immunodeficient mice.	SHED aggregate	SA-Exos shuttled miRNA-26a promoted angiogenesis via TGF-β/SMAD2/3 signalling contributing to SHED aggregate-based pulp tissue regeneration	Enhanced the angiogenic ability of HUVECs and improved pulp tissue regeneration and angiogenesis.
Liu et al., 2022 [212]	HUVECs	BALB/C nude mice.	SHED	- Enhanced growth and migration of endothelial cells. - Higher expression of VEGF, let-7f-5p and miRNA-210-3p	SHED-Exos showed pro-angiogenic effect with potential in tissue regeneration engineering.
Ivica et al., 2020 [213]	Human BM-MSCs		DPSCs	Enhanced MSCs chemotaxis & proliferation.	DPSC-Exos improved hard dental tissue regeneration.
Hu et al., 2019 [214]	Human DPSCs		DPSCs	Upregulating DSP, DMP-1, ALP, and RUNX2 and TGFβ1/smads signaling pathway and downregulating LTBP1.	DPSC-Exos promoted the odontogenic differentiation of human DPSCs.
Li et al., 2021 [215]	SCs		LPS-preconditioned human DPSCs	Stimulated DSP production and mineralization.	DPSC-Exos promoted the proliferation, migration and odontogenic differentiation of SCs.
Zhuang et al., 2020 [216]	BM-MSCs	Immunodeficient mice.	SCAP	Increased gene and protein expression of DSP.	SCAP-Exos promoted dentine-pulp complex regeneration.
Shi et al., 2023 [217].	Rat DPCs	- Rat model of pulpal defect. - Subcutaneous implantation in empty premolars root and implantation in immunodeficient mice.	Human ESC-derived MSCs line	Upregulation of odontogenic differentiation markers, DSPP, and DMP1, in addition to MEPE, BMP2, OCN, TGF-β1, ALP, BMP7, and BSP. This effect was attributed to CD73/NT5E-mediated adenosine activation of AKT and ERK signaling pathways. - Promoted the formation of reparative dentin and bridge-like structure.	MSC-Exos enhanced viability, proliferation, migration, and odontogenic differentiation of DPC in vitro and enhanced pulp-dentin tissue regeneration in vivo
Huang et al., 2021 [218].	HUVECs		LPS stimulated human DPSCs.	Upregulation of VEGF and kinase-insert domain-containing receptor	DPSCs-Exos displayed potent angiogenesis promoting effect.
Chen et al., 2021 [219].	BM-MSCs	Rat pulp-less root canal model.	LPS preconditioned human DPSCs.	- Promotion of cell proliferation, migration, angiogenesis, and differentiation. - Upregulation in the expression of ALP, DSPP, Neurofascin, and VEGF. - Pulpal regeneration, in addition to mineralized tissues formation.	DPSCs-small EVs facilitated dental pulp regeneration.
Chen et al., 2022 [220]	-SCAP -HUVECs	Exosomes loading on treated dentin matrix and subcutaneous implantation in immunodeficient mice.	DPT and DPSCs of swine.	In vitro: - Promoted SCAP and HUVEC proliferation and migration. - Enhanced angiogenesis of HUVECs. - Induced SCAP odontogenic differentiation. - In vivo, induced regeneration of vascularized pulp-like tissues associated with formation of predentin-like tissue.	Pulp tissue-Exos had the potential to be applied to the treatment of pulp deficiency caused by various pulp diseases.

ALP: Alkaline phosphatase; BALB/C: Bagg Albino; BM-MSCs: Bone marrow mesenchymal stem cells; BMP: Bone morphogenic protein; BSP: Bone sialoprotein; DMP: Dentin matrix protein; DPC: Dental pulp cells; DPSCs: Dental pulp stem cells; DSP: Dentin sialoprotein; DSPP: Dentin sialophosphoprotein; DPT: Dental pulp tissues; ESC: Embryonic stem cell; EVs: Extracellular vesicles; Exos: Exosomes; HUVECs: Human umbilical vein endothelial cells; LPS: Lipopolysaccharides; LTBP1: Latent transforming growth factor beta binding protein 1; MEPE: Matrix Extracellular Phosphoglycoprotein; miRNA-210-3p: MicroRNA-210-3p; MSCs: Mesenchymal stem cells; OCN: Osteocalcin; RUNX2: Runt-related transcription factor 2; SA-Exos: SHED aggregate-derived exosomes; SCAP: Stem cells from the apical papilla; SCs: Schwann cells; SHED: Stem cells from human exfoliated deciduous teeth; TGF-β: Transforming growth factor-β; VEGF: Vascular endothelial growth factor.

## Data Availability

No new data were created or analyzed in this study.
